# Observations on the Cow-Pox

**Published:** 1802-04

**Authors:** 

**Affiliations:** Addressed to a Physician in London


### *Observations on the Cow-Pox, by* Dr. WINTERBOTTOM, *Addressed to a Physician in London.*

I RETURN you my beft thanks for the Vaccine matter which you did me the favor of fending. In upwards of 70 cafes in which I ufed it, it anfwered my warmeft wifhes. I fhall briefly notice to you the refult of a few of the cafes, as they feem to allow of fome practical inferences being deduced.

On Saturday evening, December 5, 1801, a ftout healthy child, four years old, belonging to a Captain Park of this town, was feized with an epileptic fit, which was fuppofed to be a precurfor of the eruption of Small-pox, as the difeafe prevailed in the town in a very malignant form; though the family had anxioufly guarded againft any immediate expofure to contagion. In confequence of this alarm, four children in the fame family, between the age of feven years and eight months, were next morning inoculated with fluid Cow-pock matter taken from a puftule of the eighth day. On Monday morning the child affected with convulfions had lefs fever, no eruption appeared, and being upon the whole better, though ftill very dull and inactive, it was inoculated with fluid Vaccine matter taken from the fame child from which the other had been vaccinated the day before. On Tuefday morning, one of the children that had been inoculated on Sunday was covered with a copious eruption of Variolous puftules, attended with great fever and reftleffnefs. This child, though previoufly very healthy, appeared fomewhat indifpofed on the morning it was inoculated; its fkin was hotter than ufual, and the cheeks were fuffufed with a circular patch of red. On the fifth day, the arms of the children inoculated on Sunday exhibited certain marks of activity; the punctures, two in each arm, appeared flightly red, and were beginning to affume the indented appearance of the Vaccine puftule, puftule. The child in Small-pox continued to labour under great fever and reftleffnefs: The Variolous eruption had greatly increafed, and continued very fmall and cluftered. The inoculated fpots exactly refembled thofe of the other children. On the 8th day, the Vaccine puftules were very large, and confifted of a tumid ring, of a dirty white colour, having in the centre a minute yellow fcab nearly upon a level with the ring. On the outer part of the ring, a faint blufh, fcarcely projecting beyond its circumference, was juft preceptible. The child in Small-pox continued very ill, the fever ran high; the Variolous puftules which were ftill very fmall had become confluent in many parts, particularly on the face, and in confequence of pneumonic inflammation, a large blifter was applied to the cheft. The Vaccine puftules were almoft three times larger than any of the Small-pox, and refembled thofe of the other children in every refpect, except in not having the leaft appearance of efflorefcence which was alfo wanting at the bafe of the Variolous puftules. On this day, the child which took the fit, was inoculated a fecond time with fluid Vaccine matter taken from the arm of the youngeft child. The firft inoculation had entirely failed, though the matter fhewed its activity by caufing the punctured parts to appear very red and tumid on the fecond day; the inflammation continued to increafe until the fourth day more rapidly than in the other four children, during which time fhe continued liftlefs and difpirited, but after this period the punctures fcabbed over and fhe regained her ufual fpirits. On the ninth day the eldeft child complained of pain in the axillæ; fhe was hot, her eyes looked heavy, her head ached, and fhe vomited every thing fhe took. The Vaccine puftules were now furrounded with a fiery efflorefcence as large as a half crown, which was very painful and hard. The child in Small-pox appeared much relieved by the blifter; the refpiration was freer, the fever and reftleffnefs had abated, and the puftules had fomewhat rifen, though they ftill continued pale. The Vaccine puftules were as before, and had not the fmalleft appearance of inflammation round their bafes; the fame was obfervable of the two other vaccinated children, and the youngeft looked a little heavy and ftarted frequently.

On the tenth day the children were much the fame; the eldeft complained much of her head, vomited frequently, and the arms were very fore. The puftules on the arms of the youngeft child but one, were this day furrounded with an inflamed areola, but fhe continued well in other refpects. In the youngeft there was not the flighteft appearance of inflammation round the puftules; he continued flightly indifpofed and frequently ftarted, which was attributed to dentition.

On

On the eleventh day, the youngeſt child, inoculated on the ſixth, was covered with a copious eruption of Variolous puſtules, attended with a conſiderable degree of fever, much reſtleſſneſs, and conſtant ſtarting. The Vaccine puſtules in this child exactly reſembled thoſe of the other child in Small-Pox, and had no appearance of effloreſcence round them. The eldeſt child ſtill continued very ill, complained much of her head, and vomited every thing ſhe took, which alarmed the mother leſt ſhe alſo ſhould be affected with Small-pox; but in conſequence of the areola continuing very large and beginning to aſſume a darker red, I ventured to pronounce her ſecure. After this time, the children gradually recovered their healths, and the Vaccine puſtules ſcabbed over without producing any inconvenience.

The youngeſt child during the whole courſe of the Small-pox was unuſually reſtleſs, and was affected with ſuch violent ſtartings as almoſt reſembled convulſions. Opium neither abated theſe, nor its inceſſant cries and moans; immerſion in warm water, which was obliged to be frequently repeated, procured the only relief. The child which was a ſecond time inoculated, reſiſted once more the effects of the Vaccine virus. From this it ſeems probable that her conſtitution had ſuffered that peculiar change, during the convulſions, which is neceſſary to reſiſt the Vaccine and Variolous contagion, as ſhe lived in the ſame room and often ſlept in the ſame bed with her brothers during the whole courſe of the natural Small-pox. This appears alſo to me an additional proof that the number of perſons, who from ſome peculiarity of conſtitution are ſuppoſed capable of completely reſiſting Variolous contagion, is greatly over-rated. I have inoculated a conſiderable number of perſons with recent Vaccine virus, who poſitively declared they never had had the Small-pox; but in all theſe inſtances the reſult was agreeable to what I had foretold, the punctures inflamed for three or four days and then diſappeared.

In another family, about the ſame time as the caſes above-mentioned, a child was affected with confluent Small-pox, and on the 5th day of the eruption, three children of the ſame family were inoculated with fluid Vaccine matter. Two of the children were afterwards removed to another houſe, but the youngeſt, an infant not a month old, remained in the ſame room with the Small-pox patient, who had a ſevere diſeaſe, and recovered very ſlowly. In theſe three children the Vaccine puſtules went through the uſual courſe, and were ſurrounded with extenſive inflammations. One of the children on the eighth day complained of much uneaſineſs in the axillæ, and alſo of ſickneſs and head-ach; her ſkin was very hot, and ſhe ſeemed much indiſpoſed.

indiſpoſed. On the tenth day, about twenty or thirty puſtules reſembling varicellæ appeared upon the face and neck, which after continuing out four or five days, fell off. In another child a trifling eruption of hard dry ſpots appeared about the 10th day and ſoon diſappeared. Theſe are the only inſtances I have ſeen of puſtules during the Vaccine inoculation.

Though in the caſes above related, the ſucceſs of Vaccine inoculation was greater than I expected, ſince of eight children inoculated, five went through the diſeaſe in the uſual manner, whilſt ſurrounded with contagion of Small-pox, and of the two caſes of natural Small-pox, one only could have been prevented by the Variolous inoculation; yet the practice ought not to be followed, becauſe where there is a ſuſpicion of Small-pox contagion having been received, the moſt ſpeedy means ſhould be adopted to ſuperſede it, and this can only be done with certainty by the Variolous inoculation. I have inoculated children with Variolous matter after the diſeaſe had broke out in the family, with perfect ſucceſs; and in one caſe, the matter was taken from a perſon on the ſixth day of the eruption of a bad confluent Small-pox, to inoculate a child in the ſame houſe, which experienced the diſeaſe in its mildeſt form.

